# A single-point mutation in the rubella virus E1 glycoprotein promotes rescue of recombinant vesicular stomatitis virus

**DOI:** 10.1128/mbio.02373-23

**Published:** 2024-02-09

**Authors:** Pratyush Kumar Das, Paulina Alatriste Gonzalez, Rohit K. Jangra, Peiqi Yin, Margaret Kielian

**Affiliations:** 1 Department of Cell Biology, Albert Einstein College of Medicine, Bronx, New York, USA; 2 Department of Microbiology and Immunology, Louisiana State University Health Science Center-Shreveport, Shreveport, Louisiana, USA; Columbia University Medical College, New York, USA

**Keywords:** rubella, rubivirus, vesicular stomatitis virus, virus budding, virus fusion

## Abstract

Rubella virus (RuV) is an enveloped plus-sense RNA virus and a member of the *Rubivirus* genus. RuV infection in pregnant women can lead to miscarriage or an array of severe birth defects known as congenital rubella syndrome. Novel rubiviruses were recently discovered in various mammals, highlighting the spillover potential of other rubiviruses to humans. Many features of the rubivirus infection cycle remain unexplored. To promote the study of rubivirus biology, here, we generated replication-competent recombinant VSV-RuV (rVSV-RuV) encoding the RuV transmembrane glycoproteins E2 and E1. Sequencing of rVSV-RuV showed that the RuV glycoproteins acquired a single-point mutation W448R in the E1 transmembrane domain. The E1 W448R mutation did not detectably alter the intracellular expression, processing, glycosylation, colocalization, or dimerization of the E2 and E1 glycoproteins. Nonetheless, the mutation enhanced the incorporation of RuV E2/E1 into VSV particles, which bud from the plasma membrane rather than the RuV budding site in the Golgi. Neutralization by E1 antibodies, calcium dependence, and cell tropism were comparable between WT-RuV and either rVSV-RuV or RuV containing the E1 W448R mutation. However, the E1 W448R mutation strongly shifted the threshold for the acid pH-triggered virus fusion reaction, from pH 6.2 for the WT RuV to pH 5.5 for the mutant. These results suggest that the increased resistance of the mutant RuV E1 to acidic pH promotes the ability of viral envelope proteins to generate infectious rVSV and provide insights into the regulation of RuV fusion during virus entry and exit.

**IMPORTANCE:**

Rubella virus (RuV) infection in pregnant women can cause miscarriage or severe fetal birth defects. While a highly effective vaccine has been developed, RuV cases are still a significant problem in areas with inadequate vaccine coverage. In addition, related viruses have recently been discovered in mammals, such as bats and mice, leading to concerns about potential virus spillover to humans. To facilitate studies of RuV biology, here, we generated and characterized a replication-competent vesicular stomatitis virus encoding the RuV glycoproteins (rVSV-RuV). Sequence analysis of rVSV-RuV identified a single-point mutation in the transmembrane region of the E1 glycoprotein. While the overall properties of rVSV-RuV are similar to those of WT-RuV, the mutation caused a marked shift in the pH dependence of virus membrane fusion. Together, our studies of rVSV-RuV and the identified W448R mutation expand our understanding of rubivirus biology and provide new tools for its study.

## INTRODUCTION

Rubella virus (RuV) is an enveloped positive-sense RNA virus that is best known as a causative agent of serious birth defects in humans (1–3). While RuV infection generally produces only mild symptoms in children and adults, RuV infection in pregnant women can lead to miscarriage or congenital rubella syndrome (CRS), a collection of severe birth defects including incomplete organ development, hearing impairment, and cognitive deficiencies (2–4). Although vaccination has eliminated RuV from many parts of the world, including the Americas, worldwide, it is estimated that about 100,000 babies are born with CRS each year (5, 6). RuV is a member of the genus *Rubivirus* in the *Matonaviridae* family, with humans as the only known hosts in nature (1–3). Two new rubiviruses were recently discovered by metagenomic analyses: Ruhugu virus (RuhV), which was discovered in apparently healthy bats (7), and Rustrela virus (RusV), which was found to cause lethal encephalitis in various wild mammals and domestic cats and was also found in apparently healthy mice (7–9). These results suggested both that RuV may have had a zoonotic origin and also that rubiviruses, such as RuhV and RusV, may have the potential to spill over to humans from other animal hosts.

**Fig 1:**
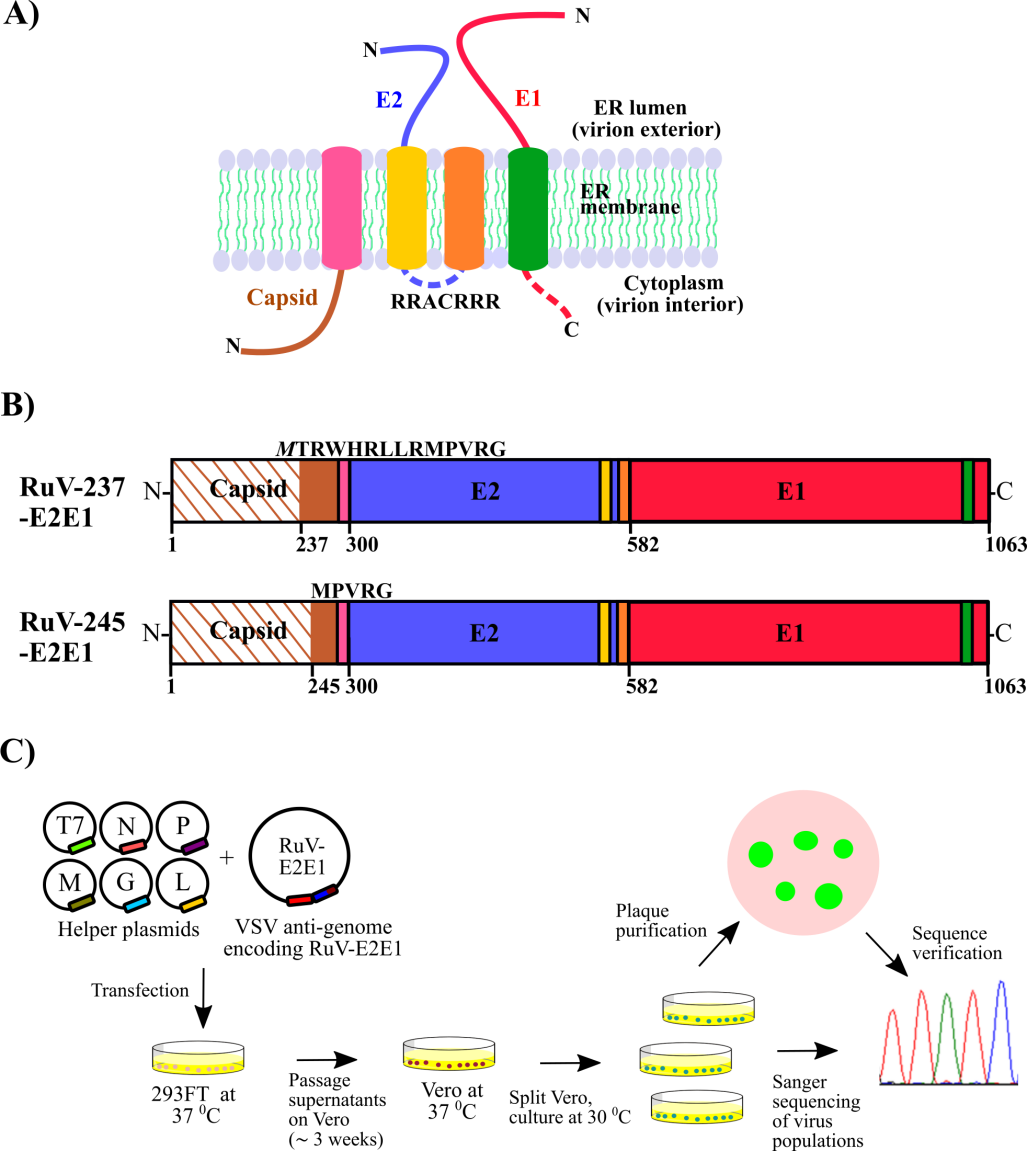
Topology and sequences of RuV structural proteins incorporated into rVSV-RuV. (**A**) Topological arrangement of the RuV structural proteins, showing Cp (brown), E2 (blue), and E1 (red) on ER or viral membrane, with the E2 SS (pink), E2 TM domain (yellow), E1 SS (orange), and E1 TM (green) in the indicated colors. An arginine-rich loop that connects the E2TM and E1SS is shown as a dotted blue line while the E1 cytoplasmic tail is depicted as a dotted red line. Redrawn from reference (3). (**B**) Schematics of the RuV E2E1 expression constructs. Features are shown as in A with polyprotein amino acid numbering at the bottom of the diagrams. The Cp C-terminal region (solid brown) harboring the E2SS (pink) marks the start site of the E2E1 expression open reading frame (ORF), while the excluded N-terminal part of Cp is shown in striped brown. Two variants of the expression cassette, termed RuV-237-E2E1 (AA 237–1063 plus an added M shown in italic) and RuV-245-E2E1 (AA 245–1063), are shown. (**C**) Rescue strategy for rVSV-RuV-E2E1. 293FT cells were transfected with pVSV antigenome plasmids encoding RuV-237-E2E1 or RuV-245-E2E1 plus the indicated helper plasmids. Supernatants from the transfected cells were repeatedly transferred onto Vero cells, which were cultured until the emergence of rVSV-RuV-E2E1.

RuV particles are rather pleomorphic and can be cylindrical or irregular in shape (2, 3). The particles contain an inner nucleocapsid core composed of the capsid protein (Cp) and the ~10 kb genomic RNA. The core is enveloped by a lipid bilayer studded with heterodimers of the E2/E1 transmembrane (TM) glycoproteins (3, 10–12). The RuV structural proteins are translated from a subgenomic RNA as a polyprotein that is cleaved cotranslationally in the endoplasmic reticulum (ER) by signal peptidase, generating Cp, E2, and E1 (2, 3, 13) (Fig. 1A). The signal sequence (SS) for E2 is retained at the Cp C-terminus and confers Cp membrane binding (14, 15), and the SS for E1 is retained at the C terminus of E2 (2, 3, 16). E2/E1 dimerizes in the ER and is cotransported to the Golgi where RuV buds (17, 18). A Golgi targeting signal was mapped to the E2TM (19).

RuV infects cells by endocytic uptake and low pH-triggered fusion in early endosomes (3, 20). E1 is the RuV membrane fusion protein and the principal target of neutralizing antibodies. The postfusion structure of E1 (21) shows that it is a class-II membrane fusion protein with two fusion loops that interact with calcium; calcium was shown to be essential for RuV fusion and infection (20, 22). RuV fusion and infectivity are inactivated by low pH, and E1 conversion to the postfusion form is irreversible (22). Following the virus budding into the Golgi, it is unclear what protects RuV from premature low pH-triggered fusion during virus exit. While morphological changes suggest that the virus undergoes maturation in the secretory pathway, the molecular mechanism of such maturation and its possible role in pH protection during virus exit are unknown (3, 18).

Vesicular stomatitis virus (VSV) is a nonsegmented negative-sense RNA virus in the *Rhabdoviridae* family (23). The VSV reverse genetic system has been effectively adapted to express and incorporate foreign glycoproteins into VSV particles (24–26). Strategies based on either replication-competent VSV (rVSV) or single-cycle pseudotyped-VSV (psVSV) allow the generation of viral particles in which the VSV G membrane protein has been replaced with heterologous glycoproteins (26). VSV particles pseudotyped with RuV glycoproteins were successfully used to test RuV cell tropism (27). However, the infectivity of psVSV pseudotyped with RuV E2/E1 was only a log higher than the psVSV without any viral glycoprotein (27). Similarly, lentiviruses pseudotyped with RuV glycoproteins produce very low levels of infectious particles (28). One challenge in these approaches may be that VSV and lentiviruses bud from the plasma membrane (PM) (29, 30), while RuV buds into the Golgi. Thus this budding site would require that the RuV envelope proteins transit through the low pH exocytic environment (31) prior to assembling onto VSV particles at the PM.

In this study, we set out to generate rVSV in which the G protein sequence was replaced with the sequence encoding the RuV E2/E1 glycoproteins. Sequencing of the rescued virus identified a W448R mutation in the E1 TM domain, a region reported to act as an ER retention signal (32). When provided in *trans*, E2/E1 carrying this mutation promoted psVSV infectivity and E2/E1 incorporation without increasing the levels of E2/E1 at the PM. Studies with rVSV and RuV showed that the mutation did not significantly affect virus cell tropism, Ca^2+^ dependence, neutralization by E1-specific antibodies, or RuV growth. However, in both rVSV and RuV, the mutation strongly shifted the fusion threshold to a more acidic pH. Taken together, our results suggest that this alteration in pH dependence promotes the ability of the RuV envelope proteins to generate infectious rVSV at the PM budding site.

## RESULTS

### A single-point mutation in RuV E1 rescues rVSV-RuV

To generate VSV-RuV recombinants (rVSV-RuV), we engineered the VSV antigenome plasmid to encode the RuV E2/E1 envelope proteins in place of the native VSV glycoprotein G. The constructs were designed to include a C-terminal region of the RuV Cp containing the E2 SS, followed by E2/E1. Two antigenome plasmids were produced: pVSV-RuV-237-E2E1 or pVSV-RuV-245-E2E1, with the Cp protein truncated at residue 237 or 245, respectively (Fig. 1A and B). To generate rVSV-RuV (26, 33, 34), we then cotransfected 293FT cells with either of the antigenome plasmids plus VSV helper plasmids (Fig. 1C). Supernatants from both sets of 293FT cells were repeatedly transferred onto naïve Vero cells. Approximately 3–4 weeks after transfection, Vero cells that received the supernatants from 293FT cells transfected with pVSV-RuV-245-E2E1 but not pVSV-RuV-237-E2E1 showed wide-spread expression of the enhanced green fluorescent protein (eGFP) reporter, indicating successful propagation of rVSV-RuV (Fig. 1C). We harvested the supernatants as the P0 stock of rVSV-RuV, sequenced the glycoprotein region of the virus population following RT-PCR, and identified a single mutation, W448R in the E1 TM domain, a region previously shown to contain an ER retention sequence (3, 32). The P0 harvest was used for preparing a plaque-purified stock of rVSV-RuV, which was sequence-verified and used for further experiments (Fig. 1C).

**Fig 2:**
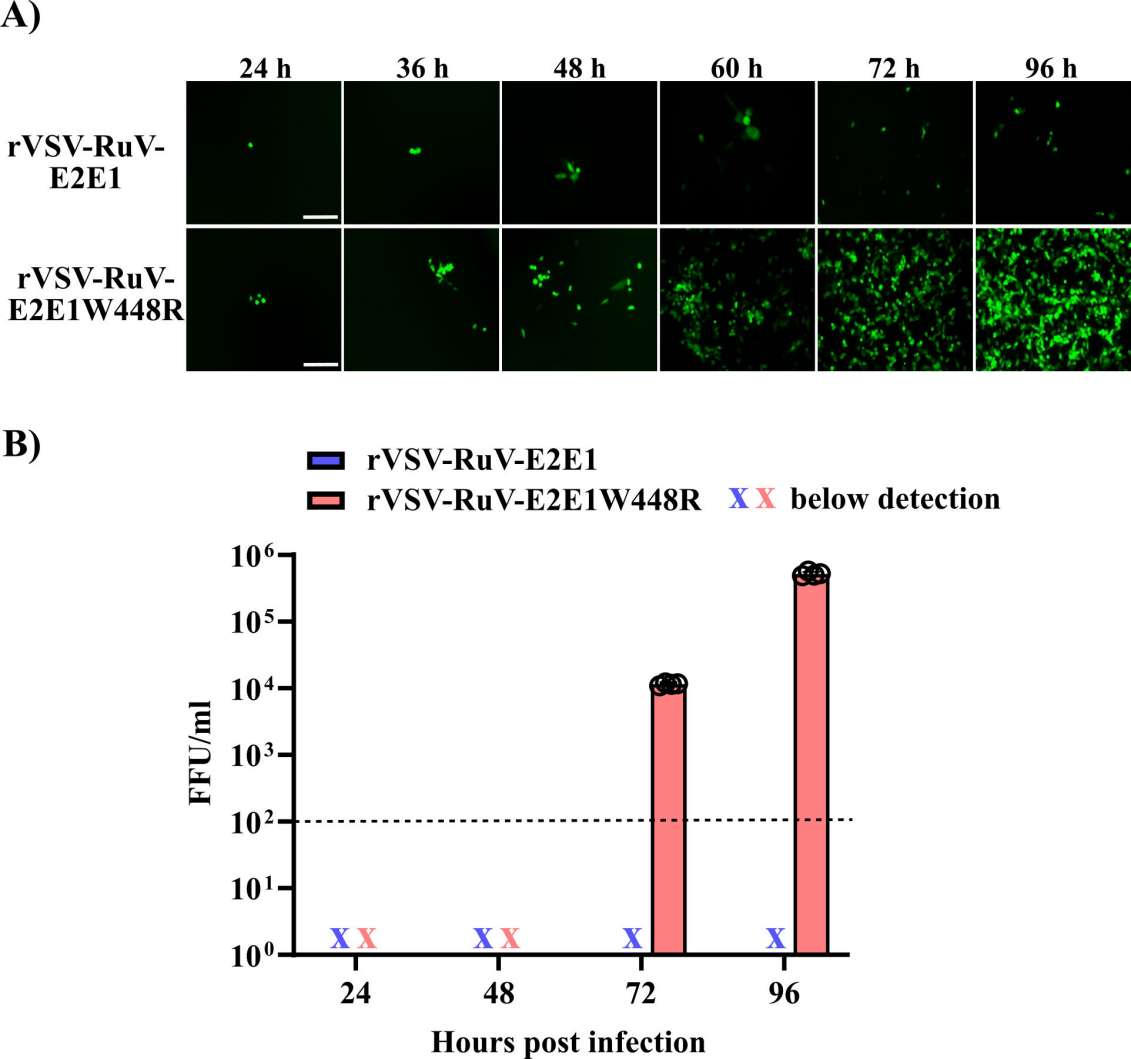
E1 W448R mutation promotes rVSV-RuV rescue. (**A and B**) Growth of rVSV-RuV-E2E1 and rVSV-RuV-E2E1W448R. 293FT cells were cotransfected with helper plasmids plus the pVSV antigenome plasmid encoding either the WT E2E1 or the mutant E2E1W448R and incubated for 5 days. Supernatants were then used to infect Vero cells. (**A**) Representative images of eGFP reporter expression in Vero cells at the indicated times post-infection (scale bar, 200 µm). (**B**) The culture supernatants from infected Vero cells were collected at the indicated timepoints and titered on Vero cells by focus-forming assay. Data shown are the mean ± SD of four independent experiments with open circles showing results from each experiment. The limit of virus detection is shown as a dotted line.

To test if the E1 W448R mutation was directly and solely responsible for the rescue of rVSV-RuV, we engineered this mutation *de novo* into the pVSV-RuV-245-E2E1 antigenome plasmid. We then transfected 293FT cells with the original pVSV-RuV-245-E2E1 or the pVSV-RuV-245-E2E1W448R mutant antigenome plasmid plus the helper plasmids, as described above. Culture supernatants were collected 5 days post-transfection and transferred onto Vero cells. At the indicated time points, Vero cell infection was monitored by imaging the eGFP signal (Fig. 2A), and culture supernatants were collected for virus titration. Both the RuV-E2E1 and RuV-E2E1W448R mutant samples produced a small number of eGFP-positive Vero cells at 24 h post-infection. While no spread of virus infection was observed in Vero cells receiving the RuV-E2E1 sample (Fig. 2A top panels), increasing infection by the RuV-E2E1W448R mutant sample was observed starting at 48 h post-infection (Fig. 2A bottom panels). The virus titer from the E1 W448R mutant sample also gradually increased, reaching 10^6^ focus-forming units (FFU)/mL at 96 h after Vero cell infection (Fig. 2B). In contrast, no infectious virus was detected from the WT sample at any of the tested time points (Fig. 2B). Thus, the E1 W448R mutation alone was key to the successful rescue of rVSV-RuV.

### Effect of E1 W448R on envelope protein expression, intracellular transport, and localization

**Fig 3:**
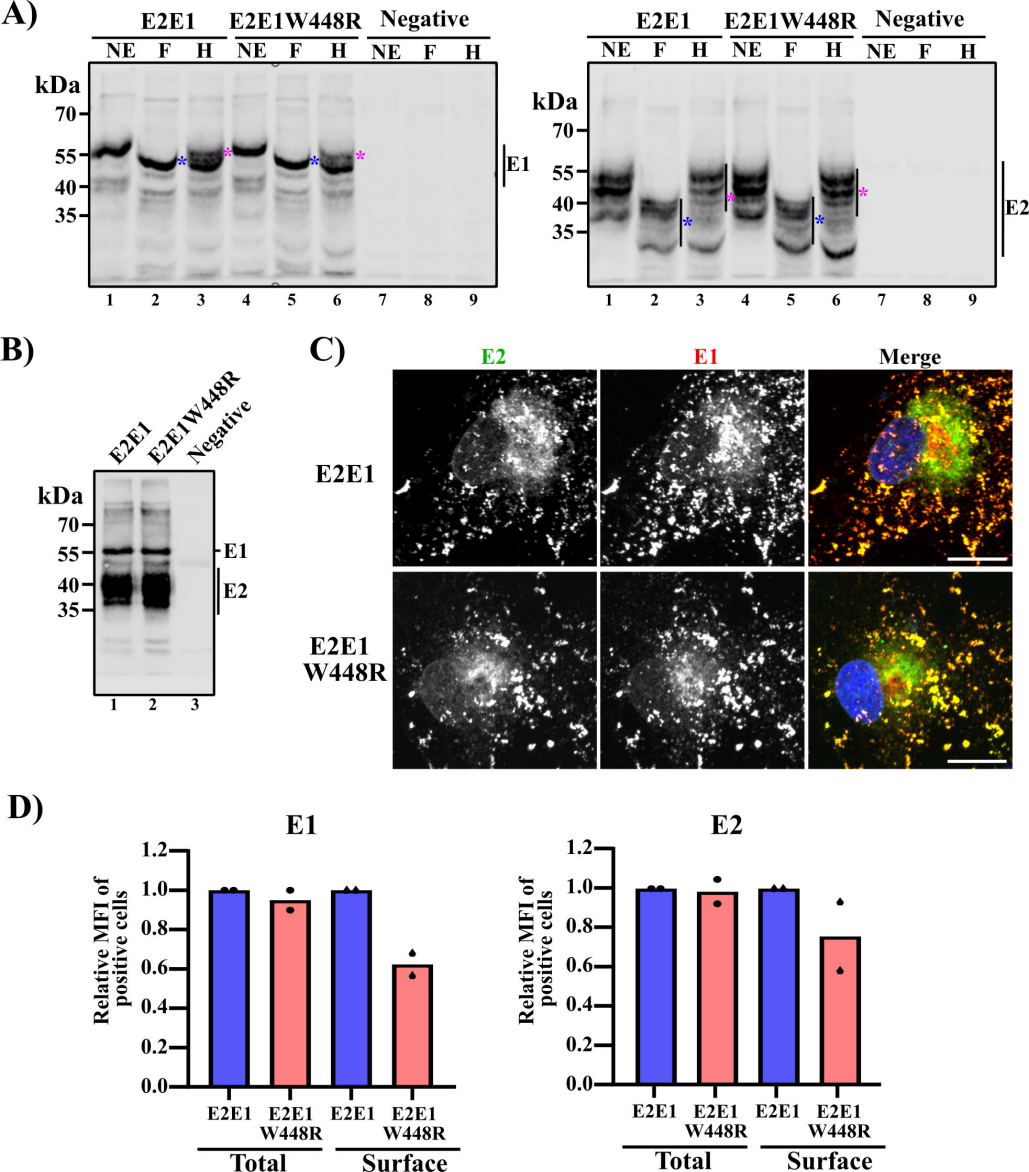
Effects of E1 W448R on the properties of expressed RuV E2/E1. (**A–D**) Vero cells were transfected with constructs expressing either the RuV WT E2E1 or the E2E1W448R mutant and analyzed as follows: (**A and B**) Cell lysates were prepared at 48 h post-transfection. (**A**) Cells were tested for E2/E1 glycosylation. Lysates were treated with no enzyme (NE), PNGaseF (**F**), or EndoH (**H**) and analyzed by WB with RuV pAb (left panel) or E2 mAb (right panel). PNGaseF-sensitive and EndoH-resistant forms of E1 and E2 are marked by blue or magenta asterisks, respectively. (**B**) E2/E1 heterodimer formation was analyzed by coimmunoprecipitation. Samples were precipitated with E1 mAb and analyzed by WB using RuV pAb to detect E1 and E2. (**C**) Colocalization of E2 and E1 was analyzed at 48 h post-transfection by staining with E2 and E1 mAb and corresponding isotype-specific secondary antibodies. Nuclei were stained with Hoechst-33342. Images were acquired using confocal microscopy and are representative examples of two independent experiments (scale bar, 10 µm). (**D**) Total and cell surface expression of E1 and E2. Cells were harvested at 24 h post-transfection, stained with antibodies against E1 or E2 under permeabilized (total) or nonpermeabilized (surface) conditions, and analyzed by flow cytometry. The relative mean fluorescence intensities (MFIs) of positive cells are shown and represent the expression of E1 or E2 relative to WT, which was set as 1. Bar graphs show the mean of two independent experiments, with individual results shown as points.

We used transient expression of the WT E2E1 and mutant E2E1W448R envelope proteins to evaluate their properties in the absence of virus replication, other viral proteins, and virus budding. Expression plasmids were transfected into Vero cells, and cell lysates were harvested at 48 h post-transfection. Western blot (WB) analysis revealed that E1 W448R does not affect steady-state expression of the E2/E1 glycoproteins (Fig. 3A). Treatment of the lysates with PNGase F and Endoglycosidase H (Endo H) (35) showed that the mutation does not detectably affect glycosylation or transport to the Golgi as defined by acquisition of Endo H resistance (Fig. 3A). Coimmunoprecipitation analyses revealed comparable E2/E1 heterodimer formation (Fig. 3B).

To analyze the subcellular localization of E2/E1 proteins, Vero cells were transfected with the WT or mutant expression constructs, fixed and permeabilized at 48 h post-transfection, and analyzed by immunofluorescence using mAbs against E2 and E1. Confocal microscopy showed that both samples had comparable colocalization of the E2 and E1 glycoproteins in the perinuclear region (Fig. 3C), consistent with the previously observed Golgi localization of the RuV envelope proteins (36). Thus, expression, glycosylation, dimerization, and intracellular localization of RuV E2/E1 were not detectably affected by the E1 W448R mutation.

Since RuV buds into the Golgi compartment (2, 3, 18) and VSV buds from the cell surface (30), we hypothesized that the E1 W448R mutation might promote E2/E1 incorporation into budding VSV particles by increasing their steady-state expression on the cell surface. To test this directly, Vero cells were transfected with WT or mutant expression plasmids, harvested after 24 h, stained with mAb against E2 or E1 under permeabilized (total) or nonpermeabilized (cell surface) conditions, and analyzed by flow cytometry. The results showed comparable total levels of E2 and E1 between WT and mutant-expressing cells (Fig. 3D), in agreement with the WB analysis (Fig. 3A). However, the results showed that the E1 W448R mutation actually caused a decrease in the cell surface levels of E2 and E1 (Fig. 3D).

### E1 W448R increases E2/E1 incorporation and psVSV infectivity

**Fig 4:**
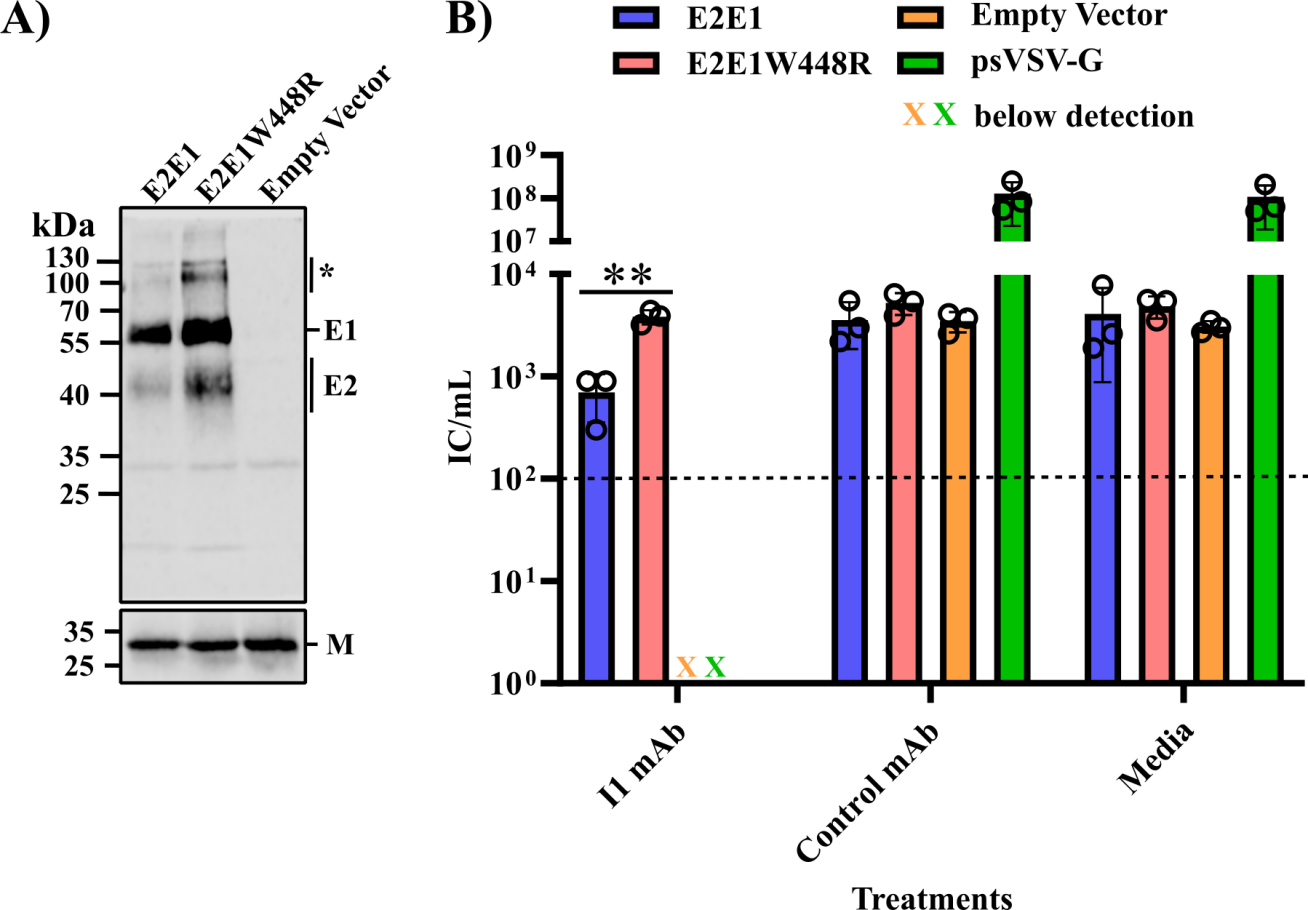
Effect of E1 W448R mutation on E2/E1 incorporation and infectivity of VSV pseudo particles. 293FT cells were transfected with an empty vector or with expression constructs for WT or E1 W448R versions of RuV E2/E1. Expressing cells were infected with psVSV-G, a single-cycle VSV lacking the G gene but carrying VSV G protein. After infection, cells were washed to remove residual inoculum and cultured for 48 h. (**A**) VSV particles in the culture supernatant were pelleted and analyzed by WB with RuV pAb and a mAb to the VSV matrix (**M**) protein. An E2/E1 population resistant to reduction and boiling is marked by an asterisk. (**B**) The infectivity of psVSV produced from cells transfected as indicated was measured by infectious center assay on Vero cells in the presence of the I1 mAb against VSV G, control mAb, or culture media alone. A psVSV-G single-cycle virus stock was used as a control for I1 neutralization. The bar graph represents the mean ± SD of three independent experiments, with open circles showing the results of individual experiments. The limit of virus detection is shown as a dotted line. Statistical analyses were performed using unpaired *t*-test. **, *P* < 0.01.

Alternatively, the E1 W448R mutation might have increased E2/E1 incorporation and/or virus infectivity as compared to the WT glycoprotein. To investigate this, 293FT cells were transfected with expression plasmids for the WT or mutant E2/E1, or with an empty expression plasmid. At 48 h post-transfection, the cells were infected with single cycle psVSV-G, which did not encode G but was pseudotyped with G by production in G-expressing cells (37). Cells were then washed extensively to remove residual inoculum and incubated for 48 h. The culture media were harvested, and equal volumes of each sample were pelleted and analyzed by WB using RuV pAb and VSV-M mAb. Production of psVSV particles, as detected by blotting for M protein, was comparable between the three samples (Fig. 4A), consistent with the known G protein-independent budding of rhabdoviruses (38, 39). We set the ratio of E1/M as 1 for WT and compared with that of the mutant. In three independent experiments, the mutant E1/M ratio was 2.6, 1.7, and 1.3 with a mean of 1.9 (Fig. 4A). We reproducibly observed a slower-migrating E2/E1 band that was resistant to SDS-denaturation and reduction, and this band was also significantly more abundant for the mutant (Fig. 4A). Thus, the E1 W448R mutation increased incorporation of the RuV glycoproteins onto psVSV particles.

Parallel aliquots of the culture media were titered on Vero cells. Media from cells transfected with the empty vector contained some infectious virus, indicating the presence of residual virus from the original inoculum (Fig. 4B). To remove this background, we incubated the culture media with I1, a neutralizing mAb against the VSV G protein (40), or with a control mAb or growth media. I1 was found to efficiently neutralize psVSV-G, specifically reducing the titer from 10^8^ IC/mL to below the limit of detection (Fig. 4B). The media sample from empty vector-transfected cells was also completely neutralized by I1 (Fig. 4B). Under these conditions, the infectivity of psVSV pseudotyped with E2/E1W448R was significantly higher than that of psVSV pseudotyped with WT E2/E1 (Fig. 4B). Together, our results suggest that the E1 W448R mutation improves RuV E2/E1 incorporation into VSV particles and their infectivity.

### RuV growth is not affected by E1 W448R

**Fig 5:**
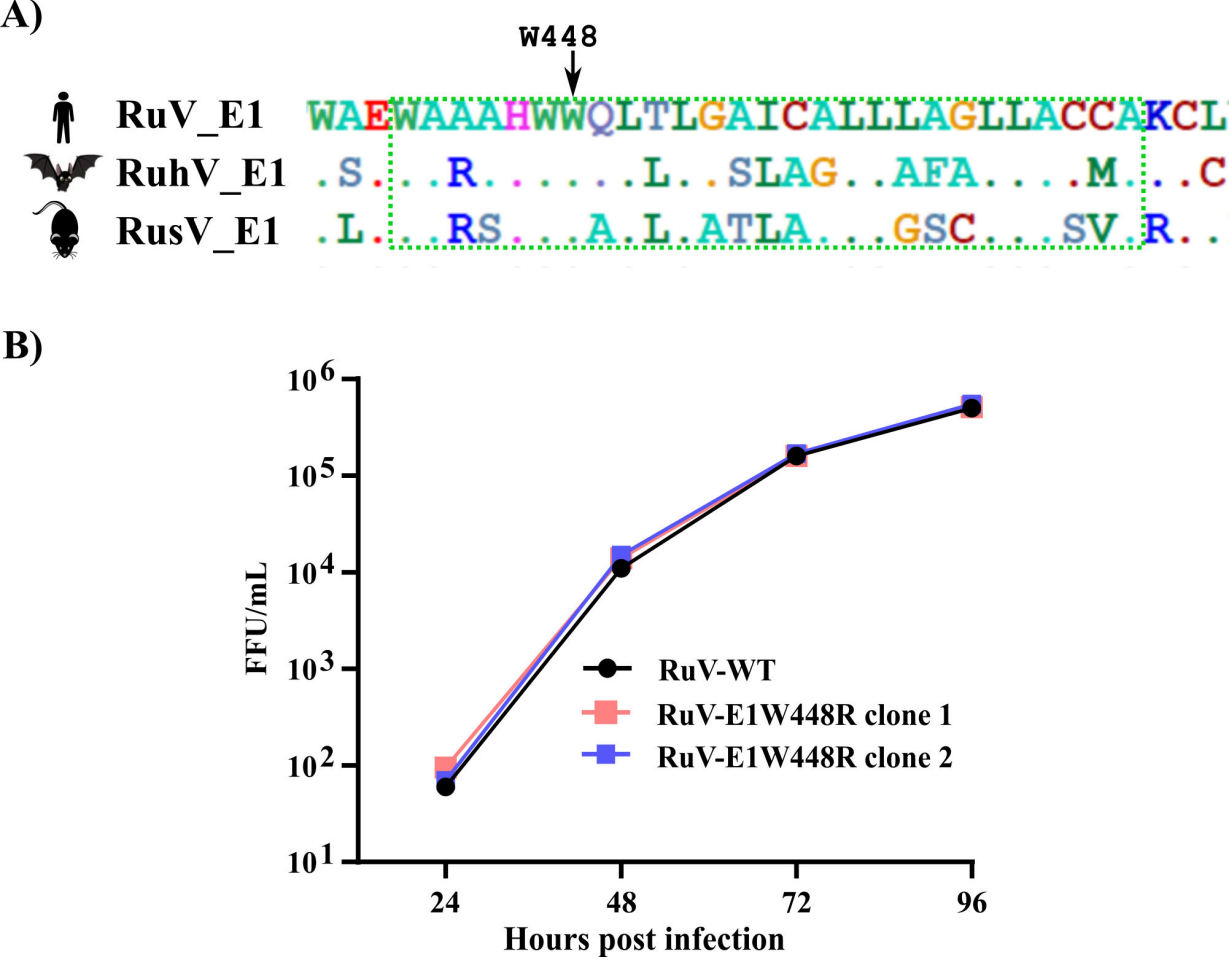
Conservation of E1 W448 and effect of mutation on RuV growth. (**A**) Amino acid sequence alignment of the E1TM region from RuV (gene bank ID P08563), RuhV (gene bank ID QKO01647.1), and RusV (gene bank ID QKO01649.2), with the putative TM domain (21, 41) indicated as a dotted green box. The natural reservoir for each virus is indicated on the left. The site of the E1 W448R mutation is indicated above (RuV E1 numbering). (**B**) Vero cells were inoculated at multiplicity of infection (MOI) = 0.01 FFU/cell with virus stocks generated from the RuV-WT infectious clone or from two independent infectious clones of the RuV-E1W448R mutant. Culture supernatants were harvested at the indicated time points and titered by focus-forming assay on Vero cells. The sequences of the mutant viruses were confirmed at the 96 h timepoint.

The E1 sequences of the related rubiviruses RuhV and RusV are ∼56% and 51% similar to that of RuV, respectively (7). We compared the RuV E1 TM region (21, 41) with those of RuhV and RusV. Alignment of the E1 TM regions revealed that E1W448 is conserved across the three species and is preceded by conserved H and W residues (Fig. 5A). Using the NCBI Virus website, we expanded our alignment by considering >150 rubivirus sequences (∼110 RuV, 3 RuhV, and ~40 RusV). This more extensive comparison revealed that while E1 W448 appears to be completely conserved, the preceding H and W are only loosely conserved across the rubivirus sequence space, suggesting a possible functional importance of E1 W448 in rubivirus biology.

To test the effect of the E1 W448R mutation on authentic RuV, this substitution was introduced into the pBRM33 infectious clone of the RuV M33 strain (41). Viral RNAs were produced by *in vitro* transcription of the RuV-WT clone and two independent mutant clones and electroporated into BHK-21/WI-2 cells (22). Culture supernatants were harvested at 48 and 72 h as the P0-48 and P0-72 stocks. Sequence analysis of the P0-48 stocks of both mutant clones confirmed the presence of the E1 W448R substitution and the absence of additional mutations in the RuV structural protein ORF. Multicycle growth curves were then performed by infecting Vero cells with P0-72 virus stock at a low MOI (0.01 FFU/cell). The results showed that both clones of RuV-E1W448R have similar growth kinetics as those of RuV-WT (Fig. 5B). Sequence analysis of the 96 h virus samples from these growth curves confirmed the presence of the E1 W448R mutation and the absence of additional mutations in the structural proteins. Thus, despite the high sequence conservation at this position, the E1 W448R mutation does not affect RuV growth in cell culture and is stable across several virus passages.

### Biological properties of E1W448R in rVSV and RuV

**Fig 6:**
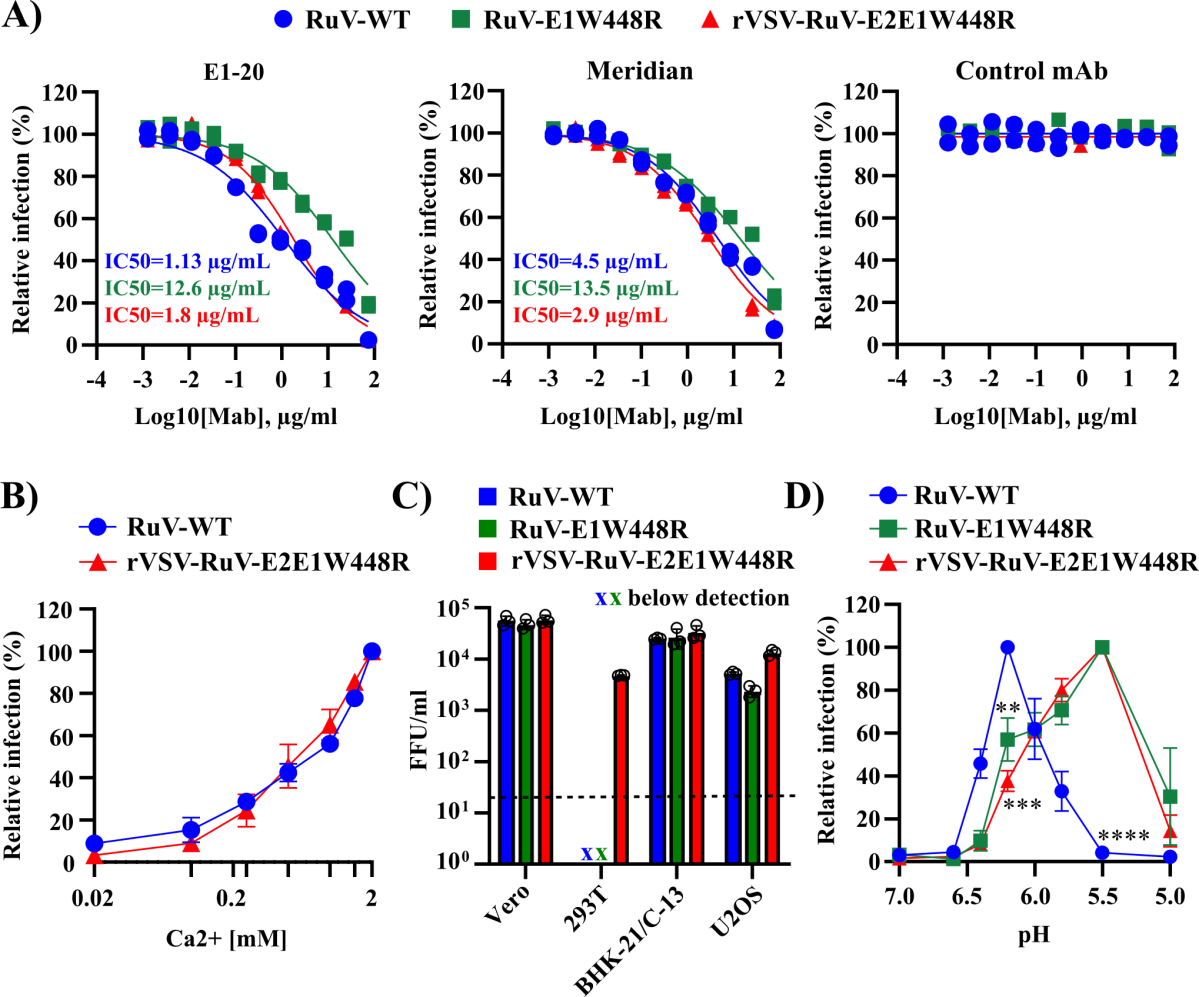
Effects of the E1 W448R mutation on the properties of E2/E1. (**A**) Neutralization of RuV-WT, RuV-E1W448R, and rVSV-RuV-E2E1W448R by RuV E1 antibodies. Viruses were incubated with the indicated concentrations of mAb E1-20 (Millipore), mAb E1 (Meridian), or the negative control mAb chCHK-152 (control mAb). Titers were determined by focus-forming assay (FFA) on Vero cells. IC_50_ values are shown as inset. (**B**) Fusion infection assay to test calcium requirement. Dilutions of RuV-WT or rVSV-RuV-E2E1W448R virus stocks were prebound to Vero cells on ice for 90 min. Cells were then incubated at 37°C for 20 min in a medium containing the indicated concentrations of CaCl_2_ and then cultured for 48 h at 37°C in a growth medium containing 20 mM NH_4_Cl to prevent secondary infection. Infected cells were scored by FFA. Infectivity was normalized to that observed at 2 mM CaCl_2_. (**C**) Cell-type dependence of primary infection of RuV-WT, RuV-E1W448R, and rVSV-RuV-E2E1W448R. Virus infectivity on the indicated cell lines was determined by FFA. The limit of detection (20 FFU/mL) is shown as a dotted line. (**D**) Characterization of the pH threshold for RuV-E1W448R and rVSV-RuV-E2E1W448R fusion. Viruses were prebound to Vero cells as in Fig. 6B, then incubated for 3 min at 37°C in the medium of the indicated pH, and cultured for 48 h at 37°C in growth medium plus 20 mM NH_4_Cl. Infection was scored by FFA and normalized to maximal fusion which was observed at pH 6.2 for RuV-WT and pH 5.5 for RuV-E1W448R and rVSV-RuV-E2E1W448R. Graphs in A show individual data points from two independent experiments. Data in B, C, and D represent the mean ± SD of three independent experiments, with the open circles in C showing the results from each experiment. Statistical analyses were carried out by one-way ANOVA with Dunnett’s multiple comparisons test. ****, *P* < 0.0001; ***, *P* < 0.001; **, *P* < 0.01.

We then tested the effects of the E1W448R mutation in the context of RuV and the VSV-RuV recombinant. The antibody response to RuV E1 has been reported to play an essential role in long-term immunity to RuV (42, 43). An E1 region from residues 223–239 is the binding site for E1-20, a potent neutralizing Ab (44). The generation of antibodies to this site strongly correlates with vaccine protection and disease convalescence in humans (43). Structural studies indicate that Abs that bind this site would prevent E1 trimerization and membrane fusion (21). We compared neutralization of RuV-WT, RuV-E1W448R, and rVSV-RuV-E2E1W448R by either E1-20 (MilliporeSigma), a similar E1 mAb from Meridian Bioscience, or a control mAb (44, 45). All three viruses were neutralized by the 2 mAbs to RuV E1 and not by the control mAb (Fig. 6A). The IC_50_ values for neutralization of RuV-WT or rVSV-RuV-E2E1W448R were between ~1 and 5 µg/mL for both mAbs. The IC_50_ for neutralization of RuV-E1W448R was higher for both mAbs, ~13 µg/mL. These results indicate that this important E1 epitope is recognized in all three viruses but may be somewhat less accessible in RuV-E1W448R.

RuV fusion is strictly dependent on Ca^2+^, which binds to conserved Asn and Asp residues in the E1 fusion loops (20–22). In the absence of Ca^2+^, E1 does not insert into the target membrane, and fusion and infection are blocked. We tested if Ca^2+^ was also required for fusion and infection of the rescued rVSV-RuV-E2E1W448R (Fig. 6B). The results showed that infection by both RuV-WT and rVSV-RuV-E2E1W448R was strongly and comparably dependent on Ca^2+^, with maximal infection occurring at a concentration of ~2 mM, in agreement with prior results.

Although humans are the only known host in nature, RuV has a wider tropism in cell culture (2, 3, 27). We compared the susceptibility of monkey, hamster, and human cell lines to RuV-WT, RuV-E1W448R, and rVSV-RuV-E2E1W448R infection. Infection of Vero, BHK-21/C-13, and U-2 OS cells was relatively efficient and broadly comparable among the three viruses (Fig. 6C). Prior reports indicated that HEK 293T cells were relatively resistant to RuV infection (46). While all three viruses showed reduced infection on HEK 293T cells, infection by rVSV-RuV-E2E1W448R was more efficient than for either RuV-WT or RuV-E1W448R (Fig. 6C).

RuV fusion is low pH-dependent, with maximal fusion observed at ~pH 6.2 (22). We used a fusion infection assay to compare the pH dependence of rVSV-RuV-E2E1W448R and RuV-E1W448R with that of RuV-WT. Viruses were prebound to Vero cells on ice, treated with buffers of varying pH for 3 min at 37°C to trigger fusion with the PM, and the resultant virus infection was quantitated (Fig. 6D). Maximal fusion of RuV-WT was observed at pH ~6.2 and rapidly declined due to virus inactivation at low pH, as previously observed (22). In contrast, both rVSV-RuV-E2E1W448R and RuV-E1W448R showed maximal fusion at ~pH 5.5 (Fig. 6D). Fusion of either virus carrying the E1 W448R mutation showed a relatively broad pH dependence and little inactivation until pH 5.0 (Fig. 6D). Thus, the E1 W448R mutation conferred a difference of ~0.7 pH units in the fusion maximum when incorporated into either rVSV-RuV-E2E1 or RuV.

### Low pH-induced conformational change of WT and mutant E1

**Fig 7:**
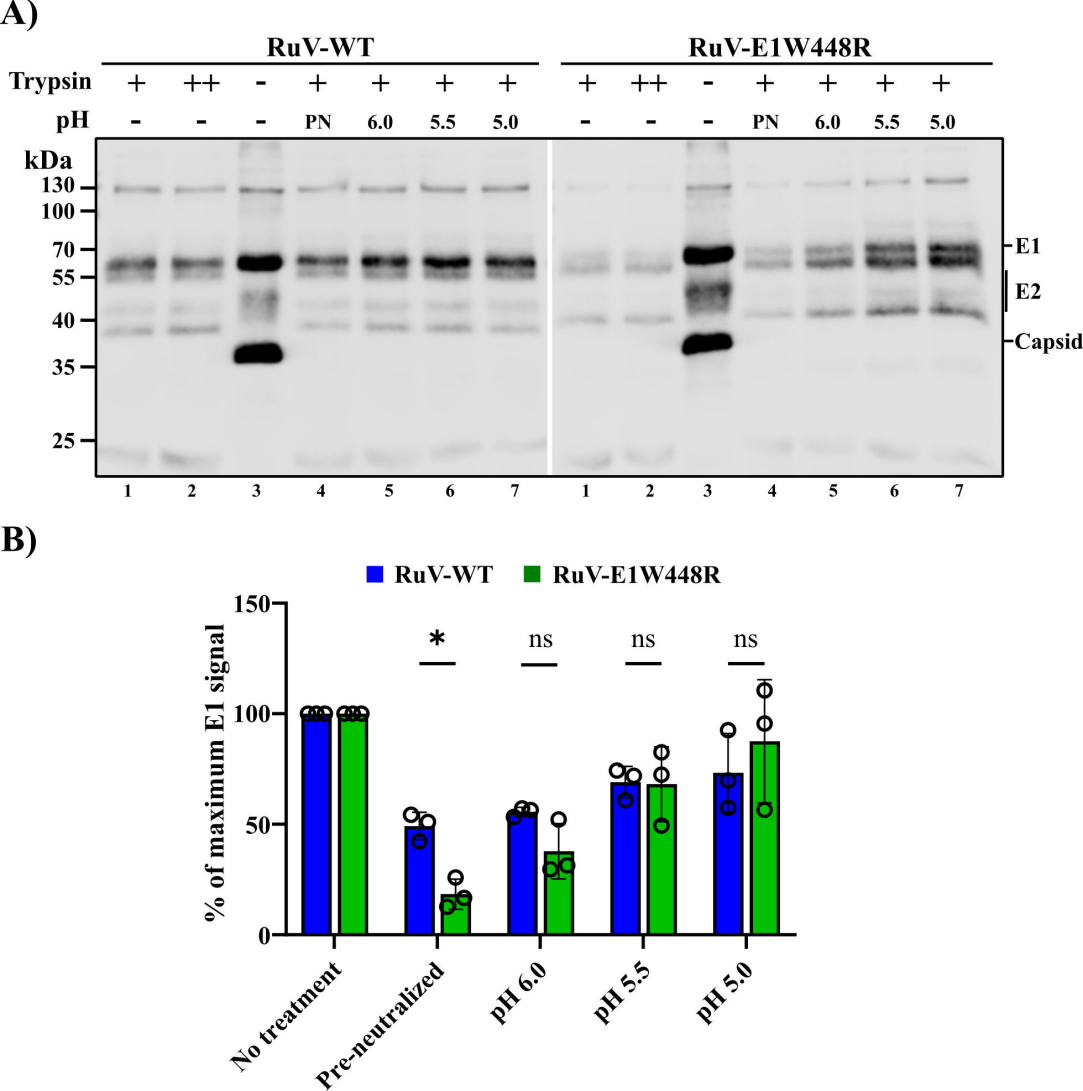
pH dependence of generation of trypsin-resistant E1. (**A**) WT or E1 W448R RuV preparations were either pre-neutralized (PN, treated with a mixture of acetic acid and HEPES mimicking the end stage of pH 5.0 treatment) or incubated at the indicated pH for 10 min at 37°C, adjusted to neutral pH, solubilized with Triton X-100, and digested for 30 min at 37°C with 125 µg trypsin/mL (+ samples). In parallel, equivalent aliquots of non-pH treated virus were incubated with 125 µg trypsin/mL (+), 200 µg trypsin/mL (++), or no trypsin (−). Samples were analyzed by SDS-PAGE and WB with RuV pAb. The white space between the WT and W448R panels indicates two different gels from the same experiment that were aligned. The data shown are representative of three independent experiments. (**B**) Quantitation of E1 trypsin sensitivity from samples prepared as in panel 7A. The E1 signal of no treatment samples (not treated with pH or trypsin, lanes 3 in panel A) was set to 100%. The trypsin-treated pre-neutralized samples (lanes 4 panel A) and pH-treated samples (lanes 5–7 in panel A) are shown relative to the no treatment samples. Data shown are the mean ± SD of three independent experiments, with individual data points shown as open circles. Statistical analyses were carried out by unpaired *t*-test. *, *P* ≤ 0.05; ns, not significant.

Low pH triggers the rearrangement of RuV E1 to the postfusion homotrimer, resulting in its increased resistance to trypsin digestion (22, 47). To test the effect of W448R on this E1 conformational change, we incubated WT or mutant RuV at the indicated pH and then digested with 125 µg trypsin/mL (Fig. 7A and B). Both the WT and mutant E1 proteins showed an increase in trypsin resistance after low pH treatment. However, control virus samples that were untreated or incubated under pre-neutralized conditions showed that WT E1 was already significantly more (40%) trypsin-resistant than mutant E1. This difference was observed even after digestion with 200 µg trypsin/mL (Fig. 7A). These results suggest that WT E1 is more sensitive to low pH exposure during virus transit through the secretory pathway and thus contains more trypsin-resistant E1 than the mutant RuV.

## DISCUSSION

Here, we describe the first successful rescue of a replication-competent rVSV encoding the RuV E2/E1 glycoproteins. This recombinant virus contained a single amino acid (AA) substitution, W448R, in the E1 TM domain. The properties of rVSV-RuV-E2E1W448R were generally comparable to those of WT RuV, showing similar localization and processing of E2/E1, virus neutralization by E1 mAbs, and virus Ca^2+^ dependence and cell tropism. However, the E1 W448R mutation markedly shifted the pH dependence of membrane fusion in either the rVSV or RuV context, from the WT RuV pH maximum of pH 6.2 to the mutant pH maximum of pH 5.5. RuV-E1W448R showed growth kinetics similar to those of WT RuV, and the mutation was stable over several passages in cell culture. We note, however, that E1 W448 is highly conserved across RuV and other rubiviruses. This stability in nature thus suggests evolutionary constraints that may reflect E1’s roles *in vivo*.

VSV was previously pseudotyped with RuV glycoproteins, albeit with relatively low efficiency (27). Successful pseudotyping occurred via the expression of the complete capsid protein along with E2/E1. Under these conditions, noninfectious RuV virus-like particles (VLPs) are produced along with the VSV-RuV pseudotypes (27). To avoid VLP production, we considered previous E2/E1 expression constructs (48) and recent structural and bioinformatic data (12, 49). We truncated Cp sufficiently to retain the E2 SS-mediated glycoprotein translocation without supporting VLP production (Fig. 1A and B). Successful rVSV-RuV rescue occurred from the shorter (RuV-245-E2E1) of our two capsid truncations.

Recombinant VSV surrogates could be a strategy to characterize novel rubiviruses, and sequence alignments show considerable conservation across the rubivirus structural proteins (3, 7). However, our attempts to rescue rVSV-RuhV and rVSV-RusV using similar structural protein constructs were unsuccessful, even if engineered with an E1W448R mutation (data not shown). Thus, while our work provides proof of concept to inform the study of other rubiviruses using rVSV, further work is needed to determine how broadly applicable this strategy could be.

Previous RuV studies showed that the E1 TM domain and cytoplasmic tail are required for virus or VLP transit out of the Golgi (41, 50, 51). The W448R substitution acquired by rVSV-RuV-E2E1 is within the E1 TM domain, which also harbors an ER retention signal (2, 3, 32). However, contrary to our initial hypothesis, the W448R mutation actually decreased cell surface levels of E2/E1. Results with psVSV indicate that despite lower levels at the PM, the E1 W448R mutation boosts E2/E1 incorporation and increases the infectivity of psVSV particles.

How does the E1 W448R mutation promote RuV glycoprotein incorporation and infectivity of rVSV-RuV-E2E1? Given the limits in sensitivity of the system, we cannot conclude that the increase in production of infectious rVSV-RuV-E2E1W448R is due solely to the observed increase in E2/E1 incorporation vs if there are additional increases due to the activity of the mutant fusion protein. Our fusion infection assay results showed that both RuV E1W448R and rVSV-RuV-E2E1W448R have a broader pH dependence for fusion, which could increase infectivity by promoting virus fusion at other points in the endocytic pathway rather than primarily at the early endosome as observed for WT RuV (20). The E1 postfusion structure does not include the TM domain and residue W448 (21). However, the fold-back mechanism of membrane fusion suggests that the TM domain would be adjacent to the fusion loop in the fused membrane (21, 52). Recent studies of the SARS-CoV-2 S protein reveal that the TM domain packs against the fusion loop in the postfusion structure, an interaction essential for membrane fusion (53). If such interactions occur in the RuV E1 protein during membrane fusion, they could be enhanced by substitutions in the E1 TM domain, although this seems less likely based on the substitution of tryptophan by the positively charged arginine residue. Instead, we favor a model in which the W448R substitution in the E1 TM domain stabilizes the E2-E1 dimer, which then shifts the pH dependence of the virus membrane fusion reaction. Since W448R is located within the E1 ER retention signal (32), it is also possible that the mutation decreases ER retention and increases transport rates. We speculate that such mechanisms could protect the RuV envelope proteins during transit through the low pH exocytic environment, promoting their cell surface delivery in a fusion-active state. This is in keeping with our finding that the mutant RuV has a lower proportion of E1 in the trypsin-resistant (postfusion) form.

Coronaviruses generally bud into the ERGIC, although egress through the lysosomal pathway has also been reported (54). The coronavirus S protein cytoplasmic tail contains an ER retention motif that slows S protein trafficking through the ERGIC (55, 56). Rescue of recombinant VSV bearing the S proteins from SARS-CoV-1, SARS-CoV-2, or MERS is facilitated by truncation of the ER retention signal (57–59). Given the wide use of the rVSV system, it would be interesting to determine if the rescue of such VSV-CoV recombinants or those of other viruses that bud intracellularly have features in common with those we describe for rVSV-RuV.

## MATERIALS AND METHODS

### Cells and antibodies

Vero cells were maintained in high glucose Dulbecco's Modified Eagle's Medium (DMEM) containing 1 mM sodium pyruvate and supplemented with 10% fetal bovine serum (FBS), 1% L-glutamine, and 1% penicillin-streptomycin (Pen-Strep). BHK-21/WI-2 and BHK-21/C-13 cells were cultured in high glucose DMEM supplemented with 1% L-glutamine, 1% Pen-Strep, and 10% tryptose phosphate broth and either 5% (BHK-21/WI-2) or 10% (BHK-21/C-13 cells) FBS (60). U-2 OS cells were cultured in modified McCoy’s 5A medium supplemented with 10% FBS and 1% Pen-Strep. 293FT cells and 293T cells were maintained in high glucose DMEM supplemented with 10% FBS, 1% L-glutamine, and 1% Pen-Strep.

Polyclonal Ab (pAb) to RuV (catalog no. AB1060), RuV mAb E1-20 (catalog no. MAB925), and mAb to VSV M protein (catalog no. MABF2347) were from MilliporeSigma. mAbs against RuV E1 (catalog no. C665063) and RuV E2 (catalog no. MA5-18255) were from Meridian Bioscience and Thermo Fisher, respectively. Recombinant mAb chCHKV-152 was a gift from Jonathan Lai at Einstein. Hybridoma cells secreting I1 antibody against VSV-G were a gift from Douglas Lyles. RuV mAbs E1-18, E1-20, and E2-1 were gifts from Tom Hobman.

### Plasmids and transfections

The AA sequence comprising a C-terminal portion of Cp and full-length E2 and E1 (structural protein ORF AA 237 to 1063 plus an additional M) from RuV M33 strain (GenBank: OM816674.1) was codon-optimized and synthesized by Twist Bioscience (South San Francisco, CA) and cloned into a mammalian expression vector and named pTWIST-RuV-237-E2E1 (Fig. 1B). A variant lacking 9 AA at the N-terminus of RuV-237-E2E1, which form a ß-strand in the Cp structure (12, 49), was engineered by standard PCR-based deletion mutagenesis and termed pTWIST-RuV-245-E2E1. This plasmid was then used to produce the E1 W448R substitution, generating pTWIST-RuV-245-E2E1W448R. To generate the rVSV antigenome plasmids, the ORF of the SARS-CoV-2 spike protein in the rVSV-SARS-CoV-2 antigenome plasmid (59) was replaced with the RuV sequences from pTWIST-RuV-237-E2E1, pTWIST-RuV-245-E2E1, or pTWIST-RuV-245-E2E1W448R by using MluI and NotI restriction sites, generating the antigenome pVSV-RuV-237-E2E1, pVSV-RuV-245-E2E1, and pVSV-RuV-245-E2E1W448R plasmids. The rVSV antigenome plasmid also encodes eGFP as a separate transcriptional unit (59). The rVSV-SARS-CoV-2 antigenome plasmid and helper plasmids were obtained from Kartik Chandran (33, 34, 59). The pBRM33 infectious clone of the RuV M33 strain (41) (a gift from Tom Hobman) was engineered with the E1 W448R substitution using PCR-based mutagenesis of an intermediate plasmid (pUC19_BamHI_HindIII_M33). The mutagenized fragment was ligated into pBRM33 using the BamHI and HindIII restriction sites to generate pBRM33-E1W448R. All plasmid sequences were verified by Sanger sequencing (Azenta Life Sciences).

Transfection of 293FT cells was performed using polyethylenimine (PEI MAX, Polysciences, Inc). PEI was diluted to 1 mg/mL in Milli-Q water, adjusted to pH 7.25 with NaOH, and filtered-sterilized using a 0.22 µm syringe filter. A 4:1 ratio of PEI/DNA was used for each transfection reaction. Cells were incubated for 6 h at 37°C in Opti-MEM (Gibco) media containing the PEI/DNA mixture and then maintained in high glucose DMEM supplemented with 5% FBS. Vero cells were transfected where indicated using Lipofectamine 2,000 (Invitrogen) according to the manufacturer’s instructions.

### Virus titration

Virus samples were titered on Vero cells using infectious center assays (ICA) or focus-forming assays (FFAs). Vero cells were seeded at 1.2 × 10^4^ cells/well in 96-well plates, cultured overnight, and infected with 100 µL of serial dilutions of virus samples for 4 h. The virus inocula were aspirated and replaced with 100 µL of DMEM plus 5% FBS and 20 mM NH_4_Cl or 1% carboxymethylcellulose in modified Eagle’s Medium supplemented with 2% heat-inactivated FBS and 10 mM Hepes pH 7.4. For ICA, 48 h post-infection, cells were fixed with 4% paraformaldehyde (PFA, Electron Microscopy Science) and stained with RuV pAb and fluorescently labeled secondary antibody, and infection was quantitated by fluorescence microscopy. For FFA, cells were fixed by adding 100 µL of prewarmed 1% PFA in PBS to the overlay and incubating for 1 h. Cells were washed with PBS, permeabilized with 0.1% saponin in PBS containing 0.1% bovine serum albumin (BSA), incubated with RuV pAb followed by incubation with horseradish peroxidase-conjugated rabbit anti-goat IgG (Seracare, Milford, MA). Foci were developed using TrueBlue Peroxidase substrate (Seracare) and quantified using an ImmunoSpot S6 Macroanalyzer with Biospot 7.0.9.10 software (Cellular Technologies, Shaker Heights, OH). The titer for psVSV-RuV was determined by ICA, with initial infection for 2 h and scoring of eGFP-positive cells 24 h post-infection.

### VSV-RuV rescue

The standard plasmid-based VSV reverse-genetic system was used to rescue rVSV-RuV-E2E1 (33, 34). 293FT cells were cotransfected with the pVSV-RuV-237-E2E1 or pVSV-RuV-245-E2E1 antigenome plasmids and helper plasmids expressing T7 polymerase and VSV N, P, M, G, and L. Starting at day 2 post-transfection, supernatants from the transfected cells were added to Vero cells every 24 h, and the eGFP signal was monitored by fluorescence microscopy. At 3 weeks post-transfection, the Vero cells were split, and the cultures transferred to 30°C.

Approximately 1 month after transfection, the Vero cells showed a wide-spread eGFP signal. The supernatants from two parallel plates were harvested as the P0 stock of rVSV-RuV and titered by ICA on Vero cells. RNA was extracted from the virus-containing culture media, reverse transcribed, and PCR amplified, and the sequence of the RuV structural protein region was determined. The P0 virus stock was then plaque-purified and used to generate a P1 stock termed rVSV-RuV-E2E1W448R, which was verified by sequencing and used for subsequent experiments.

For validation of the E1 W448R mutation as the driver of rescue, 293FT cells were cotransfected with pVSV-RuV-245-E2E1 or pVSV-RuV-245-E2E1W448R antigenome plasmids and helper plasmids as above. Supernatants were harvested after 5 days and used to infect naïve Vero cells. Infected Vero cells were incubated at 30°C, and the eGFP signal was monitored daily by fluorescence microscopy (Zeiss Axiovert 200M). In parallel, supernatant samples were collected at the indicated timepoints and titered by FFA.

### RuV production and growth assays

Infectious RuV RNA was generated by *in vitro* transcription (22) of the WT or mutant pBRM33 and electroporated into BHK-21/WI-2 cells. The P0 RuV stocks were harvested at 48 h postelectroporation, and the virus RNA was reverse-transcribed and the structural ORF sequenced by Sanger sequencing. For growth assays, Vero cells were seeded at 1 × 10^5^ cells/well in 12-well plates, cultured overnight, and infected with P0 stock at MOI 0.01 FFU/cell. Culture media were harvested at the indicated time points and titered by FFA. The sequence of the mutant virus harvested at 96 h post-infection was analyzed as described above.

### Glycosylation and coimmunoprecipitation assays

Vero cells were seeded at 3.2 × 10^5^ cells/well in six-well plates and incubated overnight, and duplicate wells were transfected with pTWIST-RuV-245-E2E1 or pTWIST-RuV-245-E2E1W448R. Cells were lysed 48 h post-transfection in 0.15 mL of ice-cold lysis buffer [50 mM TRIS pH 7.4, 100 mM NaCl, 1 mM EDTA, 1% TritonX-100, 1 µg pepstatin/mL, 2 µg aprotinin/mL, and 1 mM phenylmethylsulfonyl fluoride (PMSF)], and duplicate samples were combined and clarified by centrifugation. For glycosylation analysis, aliquots of lysates were denatured at 100°C for 10 min in the presence of 40 mM dithiothreitol (DTT) and 0.5% SDS and digested with PNGase F or Endoglycosydase H according to the manufacturer’s protocol (New England Biolabs). For coimmunoprecipitation, 200 µL of lysates were precleared with Protein-A agarose beads (Thermo Fisher), then incubated with E1-20 mAb for 60 min on ice. Protein-A agarose beads were added, and samples were rotated for an additional 90 min. Beads were washed three times with wash buffer (50 mM TRIS pH 7.4, 100 mM NaCl, 1 mM EDTA, 0.1% Triton X-100, and 2 µg/mL aprotinin) and resuspended in elution buffer (10 mM TRIS pH 6.8, 1 mM EDTA, 1 mM PMSF, and 2 µg aprotinin/mL) and stored at −20°C. Before gel analysis, buffer containing SDS and DTT was added to 0.5% and 40 mM, respectively, and samples were boiled at 100°C for 10 min. Samples were analyzed by SDS-PAGE and WB using RuV pAb and E2 mAb.

### Confocal microscopy

Vero cells were seeded on coverslips and transfected with pTWIST-RuV-245-E2E1 and pTWIST-RuV-245-E2E1W448R expression constructs. 48 h post-transfection cells were fixed using 4% PFA, permeabilized with 0.2% TritonX-100, stained with mAb E1-18 and E2-1 (1:50 dilution), and the appropriate isotype-specific Alexa Fluor-conjugated secondary antibody (1:2500, Thermo Fisher) and Hoechst dye (1:10,000, Thermo Fisher). Images of 15–20 randomly chosen cells for each construct were acquired using a 100× oil objective on a Nikon CSU-W1 Spinning Disk confocal microscope at the Einstein Analytical Imaging Facility. Z-stacks were acquired for the entire depth of the cell at a step size of 0.2 µm. Figures were prepared using the ImageJ QuickFigures plugin (NIH) and Inkscape.

### Flow cytometry

Vero cells were seeded at 3 × 10^5^ cells/well in six-well plates cultured overnight at 37°C, transfected with the pTWIST-RuV-245-E2E1 or pTWIST-RuV-245-E2E1W448R expression constructs, and incubated for 24 h at 30°C. Cells were harvested using Accutase (Sigma) and washed two times with staining buffer (15 mM HEPES pH 7.0 and 2% FBS in PBS). For intracellular staining, the cells were fixed with 2% PFA in PBS for 10 min at room temperature and permeabilized by 0.01% Triton in staining buffer for 10 min at room temperature. For cell surface staining, the cells were blocked in staining buffer, washed once, and stained with RuV E1-20 (1:200, MilliporeSigma) or RuV E2 (1:25, Thermo Fisher) for 40 min at 4°C. Then the cells were washed twice and stained with appropriate Alexa Fluor-conjugated secondary antibody at 1:500 for 30 min at 4°C. The cells were washed twice, postfixed with 2% PFA for 10 min at room temperature, and then washed twice with PBS. 1 × 10^4^ cells were analyzed for each sample using a BD LSR-II analyzer (BD Biosciences, San Jose, CA, USA) in the Einstein flow cytometry core. Mock-infected cells stained as above were used to delineate the gates for flow analysis (60). The flow data were processed using FlowJo 10.2 software.

### Incorporation experiments

293FT cells were seeded in 10 cm culture dishes precoated with poly-D-lysine and cultured overnight, and duplicate plates were transfected with pTWIST-RuV-245-E2E1, pTWIST-RuV-245-E2E1W448R, or empty expression vector. 48 h post-transfection cells were gently washed with DMEM and inoculated with single cycle psVSV-G [VSV with a G protein deletion, encoding the eGFP reporter and pseudotyped with G by production in G-expressing cells (37), a gift from Drs. Megan Slough and Kartik Chandran]. After a 2 h incubation, cells were washed eight times with DMEM and incubated at 30°C for 48 h, and the culture media were harvested. Aliquots were stored at −80°C for titration. Equal volumes of freshly harvested supernatants for each construct were pelleted at 20,000 rpm in an SW32Ti rotor for 2 h at 4°C. The virus pellets were resuspended in DMEM and pelleted through a 10% sucrose cushion [(wt/vol) in 50 mM Tris-HCl, 100 mM NaCl] in an SW41 rotor at 20,000 rpm for 2 h at 4°C. The virus pellets were resuspended in 100 µL buffer containing 50 mM TRIS pH 7.4, 100 mM NaCl, 1 mM PMSF, 1 ug/mL pepstatin, and 2 µg/mL aprotinin and stored at −80°C. Just before gel analysis, samples were adjusted to 0.5% SDS and 40 mM DTT and boiled for 10 min at 100°C. Samples were analyzed by SDS-gel electrophoresis and WB using RuV pAb and a mAb to VSV-M protein. For titration, samples were incubated with I1, control antibody, or media for 1 h and then titered by ICA.

### Assays of E1 W448R properties in VSV and RuV

To test virus calcium dependence, ~150 FFU of RuV-WT or rVSV-RuV-E2E1W448R was bound on ice to prechilled Vero cells for 1.5 h in calcium-free binding medium (calcium-free MEM without NaHCO_3_ plus 0.2% BSA and 10 mM Hepes pH 7.0). Unbound viruses were removed, and cells were incubated at 37°C for 20 min in a calcium-free binding medium containing the indicated concentrations of CaCl_2_. The cells were then incubated for 48 h at 30°C in a growth medium (with calcium) containing 20 mM NH_4_Cl to prevent secondary infection and scored by FFA.

To determine the pH dependence for virus fusion, 150 FFU of RuV-WT, RuV-E1W448R, or rVSV-RuV-E2E1W448R were bound on ice to prechilled Vero cells for 1.5 h in binding medium (RPMI 1640 without NaHCO_3_, plus 0.2% BSA, and 10 mM Hepes pH 7.0). Unbound viruses were removed, and cells were incubated at 37°C for 3 min in a fusion medium (binding medium plus 20 mM MES) adjusted to the indicated pH. After the pH pulse, the fusion medium was replaced with a growth medium containing 20 mM NH_4_Cl. Cells were incubated at 30°C for 48 h and scored by FFA.

To test cell tropism, Vero, U-2 OS, BHK-21/C-13, or 293T cells were seeded at 1.8 × 10^4^ cells/well in 96-well plates, cultured overnight, and inoculated with 3-fold serial dilutions of RuV-WT, RuV-E1W448R, or rVSV-RuV-E2E1W448R (starting at 6 × 10^4^ FFU/mL). 3 h post-infection, the media were replaced with 1% carboxylmethylcellulose overlay, the samples were incubated at 30°C for 48 h, and infection was scored by FFA.

### Antibody neutralization assay

Approximately 150 FFU of RuV-WT, RuV-E1W448R, or rVSV-RuV-E2E1W448R were incubated for 1 h at 37°C with 3-fold serial dilutions of the indicated mAbs (starting with 300 nM) in MEM plus 0.2% BSA and 10 mM HEPES pH 7.0. Vero cells in 96-well plates were infected with the antibody:virus complexes for 3 h, the media were replaced with 1% carboxylmethylcellulose overlay, and the samples were incubated at 30°C for 48 or 30 h (rVSV-RuV only) and scored by FFA. The number of foci in wells containing mAb was normalized to wells infected with the virus without antibody. Nonlinear regression analysis was performed, and IC_50_ was calculated using Prism 10 (GraphPad Software).

### Assay of generation of trypsin-resistant E1

P1 stocks of RuV-WT and RuV-E1W448R were prepared by infecting Vero cells with P0 stocks at an MOI of 0.1 FFU/cell. At 96 h post-infection, the supernatants were harvested and pelleted through a 10% sucrose cushion [(wt/vol) in 50 mM TRIS pH 7.4 and 100 mM NaCl] by centrifugation in an SW32Ti rotor (Beckman Coulter) at 28,000 RPM for 2 h. The virus pellets were resuspended in 10 mM MES pH 7.0, 10 mM HEPES pH 7.0, and NaCl 100 mM overnight on ice and aliquoted and stored in −80°C.

pH treatment and trypsin digestion of WT and mutant RuV were carried out as described previously (22, 47). Briefly, for each treatment, 30 µL of the resuspended virus was treated at the indicated pH for 10 min at 37°C by adding pre-calibrated volumes of 0.5 N acetic acid followed by neutralization with 1M HEPES pH 8.0. pre-neutralized samples were treated with a mixture of acetic acid and HEPES equivalent of pH 5.0 treatment. Samples were then solubilized for 10 min on ice with a final concentration of 0.9% TritonX-100 and then digested as indicated with trypsin (Sigma-Aldrich catalog no. T1426) prepared in PBS containing 0.9 mM CaCl_2_ and 0.5 mM MgCl_2_ at a final concentration of 125 or 200 µg/mL for 30 min at 37°C. Digestion was quenched by adding PMSF to a final concentration of 1 mM. 0.5% SDS and 40 mM DTT were added, and samples were heated for 5 min at 95°C and analyzed by SDS-PAGE and WB using RuV pAb.

### Statistics

All statistical analyses were carried out using GraphPad Prism 10. The specific analyses are listed in the figure legends.
